# Whole blood transcriptome profiling identifies gene expression subnetworks and a key gene characteristic of the rare type of osteomyelitis

**DOI:** 10.1016/j.bbrep.2022.101328

**Published:** 2022-08-27

**Authors:** Hiroko Yahara, Souichi Yanamoto, Miho Takahashi, Yuji Hamada, Haruo Sakamoto, Takuya Asaka, Yoshimasa Kitagawa, Kuniyasu Moridera, Kazuma Noguchi, Masaya Sugiyama, Yutaka Maruoka, Koji Yahara

**Affiliations:** a Genome Medical Science Project, Research Institute, National Center for Global Health and Medicine, Toyama, Shinjuku-ku, Tokyo, Japan; b Department of Clinical Oral Oncology, Nagasaki University Graduate School of Biomedical Sciences, Nagasaki, Japan; c Department of Oral and Maxillofacial Surgery, Tokai University Hachioji Hospital, Japan; d Department of Oral Diagnosis and Medicine, Hokkaido University Graduate School of Dental Medicine, Sapporo, Japan; e Department of Oral and Maxillofacial Surgery, Hyogo College of Medicine, Nishinomiya, Hyogo, Japan; f Department of Oral and Maxillofacial Surgery, Center Hospital of the National Center for Global Health and Medicine, Tokyo, Japan; g Antimicrobial Resistance Research Center, National Institute of Infectious Diseases, Tokyo, Japan

**Keywords:** Osteomyelitis, CNO, RNA-Seq, Gene expression, Network, Differentially regulated genes, CNO, chronic non-bacterial osteomyelitis, TPM, transcripts per million

## Abstract

Chronic non-bacterial osteomyelitis (CNO) is a rare and severe inflammatory bone disorder that can occur in the jaw. It is often associated with systemic conditions including autoimmune deficiency. Medical management of patients and establishment of a correct diagnosis are difficult as the etiology of the disease remains unknown. Therefore, little is known about the disease characteristics at the gene expression level. Here, we explored aspects of CNO based on whole blood RNA sequencing (>6 Gb per sample) of 11 patients and 9 healthy controls in Japan and on a recently developed method that is applicable to small datasets, can estimate a directed gene network, and extract a subnetwork of genes underlying patient characteristics. We identified nine subnetworks, comprising 26 differentially regulated edges and 36 genes, with the gene encoding glycophorin C (GYPC) presenting the highest discrimination ability. The expression of the gene was mostly lower in patients with CNO than in the healthy controls, suggesting an abnormal status of red cells in patients with CNO. This study enhances our understanding of CNO at the transcriptome level and further provides a framework for whole blood RNA sequencing and analysis of data obtained for a better diagnosis of the disease.

## Highlights

• We explored new aspects of chronic non-bacterial osteomyelitis (CNO) via whole-blood RNA-seq and gene network analysis.
• Nine subnetworks of genes were identified.
• A GYPC-encoding gene had the highest discrimination ability in these subnetworks.
• The expression level of the gene suggested an abnormal status of red cells in the patients.

## 1 Introduction

Osteomyelitis of the jaw is a severe inflammatory disorder affecting the bones, and the medical management of patients with osteomyelitis has been unsatisfactory owing to the unknown etiology of the disease and difficulties in establishing a correct diagnosis. Chronic non-bacterial osteomyelitis (CNO) is a rare type of chronic osteomyelitis, and some patients with CNO do not respond to conventional antibiotic therapy [1]. CNO of the jaw is associated with several systemic conditions, including autoimmune deficiency, rheumatic arthritis, chronic inflammatory bowel disease, and palmoplantar pustulosis [2].

Little is known about the dynamics of inflammatory responses in CNO or how the genetic and immunological backgrounds of patients influence the disease. A clue in this regard has been provided by a recent study [3] that aimed to overcome the limitations of traditional exon typing in the human leukocyte antigen (*HLA*) gene region, based on latest sequencing technologies [4]. The recent study simultaneously determined the alleles of all 35 HLA loci and haplotype structures of the killer cell immunoglobulin-like receptor (KIR) region and identified a specific amino acid substitution in HLA-C, in combination with the telomeric KIR genotype, which has a significantly higher frequency in the CNO population than in the control population [3].

The latest sequencing technologies can deal with the complexity of the entire HLA and KIR regions; however, they require special protocols for next-generation sequencing [5]. Elucidation of the characteristics of CNOs using general and standard protocols remains a challenge. In this milieu, we aimed to explore new aspects of CNO based on whole blood RNA sequencing (RNA-Seq) of patients with CNO and healthy controls. This technique does not require special protocols but exploits the fact that peripheral blood is highly accessible and is a reliable indicator of human health. As CNO is a rare disease, the sample size of our study was small, although the samples were collected from several university hospitals in different geographical regions in Japan. Therefore, we utilized a recently developed method that is applicable to small sample datasets (e.g., 18 samples across three groups) and can extract a subnetwork of genes underlying patient characteristics [6].

We conducted whole blood RNA-Seq of patients with CNO and healthy controls in the Japanese population and gene expression network analysis to identify biomarkers at the gene expression level that could be useful for a better diagnosis of CNO.

## 2 Materials and methods

### 2.1 Sample collection, library preparation, and RNA-Seq

We used the same diagnostic criteria as in our previous study [3]. The criteria were as follows: 1) recurrent pain and swelling; 2) radiographic appearance of a mixed pattern of sclerosis and osteolysis and uptake of scintigraphic agents, such as technetium 99 m, in the region of the jawbone; 3) little or no benefit from antibiotic treatment; and 4) increased bone resorption and deposition, and varying degrees of bone sclerosis and medullary fibrosis with no suspected malignancy. Blood samples (2.5 mL) were collected in PAXgene blood RNA tubes from 12 patients with CNO and 12 healthy controls. RNA was extracted from blood samples using the PAXgene Blood RNA Kit.

The extracted RNA was subjected to quality control (QC) by Novogene Inc., and the process involved (1) preliminary quantitation using Nanodrop, (2) analysis of RNA degradation and potential contamination using agarose gel electrophoresis, and (3) verification of RNA integrity and quantitation using Agilent 2100. After QC of the samples, 1 patient with CNO and 3 healthy controls with total RNA < 0.07 μg were excluded. The remaining samples were subjected to library preparation and RNA-Seq by Novogen Inc. Globin mRNA was removed from the total RNA using the GlobinClear Kit. mRNA was enriched using oligo(dT) beads, and rRNA was removed using the Ribo-Zero Kit. First, the mRNA was randomly fragmented by adding fragmentation buffer, and cDNA was synthesized using an mRNA template and random hexamer primers, after which a custom second-strand synthesis buffer (Illumina), dNTPs, RNase H, and DNA polymerase I were added to initiate the second-strand synthesis. Second, after a series of terminal repairs, a ligation, and sequencing adaptor ligation, a double-stranded cDNA library was constructed through size selection and PCR enrichment. QC of the libraries was conducted, with the following three steps: (1) preliminary test of the library concentration using Qubit 2.0, (2) test of the insert size using Agilent 2100, and (3) precise quantification of the effective concentration of library using qPCR.

The libraries were sequenced using Illumina sequencers after pooling according to their effective concentration and expected data volume of 6 Gb per sample.

### 2.2 Bioinformatic analysis

**Fig. 1:**
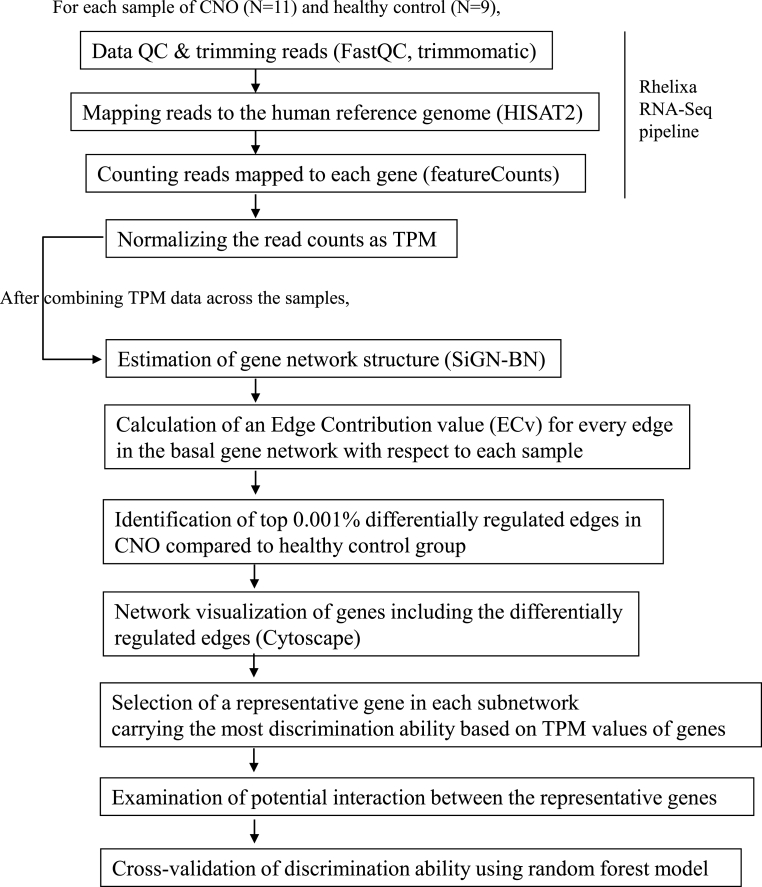
**Overview of bioinformatic analysis.** Names of software used in each step are provided in parentheses. The first three steps were implemented according to the Rhelixa RNA-Seq pipeline.

For each sample of CNO and healthy controls, data QC, mapping reads to the human reference genome, and counting reads mapped to each gene were conducted using the Rhelixa RNA-Seq pipeline, in which FastQC [7], trimmomatic [8], HISAT2 [9], and featureCounts [10] were used (Fig. 1). The read count data were normalized to transcripts per million (TPM) using R statistical software. After combining TPM data across the samples into a matrix, the gene expression network structure was estimated using a Bayesian network with the SiGN-BN program, included in SiGN [11], a collection of large-scale gene expression network estimation software. We repeated the estimation thrice and confirmed that the estimated gene expression network structure was the same. The edge contribution value (ECv) was calculated [6] using ECv software for every edge in the estimated basal network with respect to each sample. The average ECv was calculated for the CNO and healthy control groups for every edge, and the top 0.001% differentially regulated edges in terms of the extent of difference in the average ECv per group were identified in the CNO group compared with those in the healthy control group. We focused on the top 0.001% differentially regulated edges identified in the CNO group and conducted network visualization using Cytoscape [12]. A classification tree analysis based on TPM values of genes was conducted for each subnetwork of the visualized network using JMP Pro version 13 (SAS Institute, Cary, NC, USA), as well as automatic variable selection in the logistic regression model for each subnetwork using R statistical software. Potential interactions between the representative genes were also explored using the logistic regression model. Leave-one-out cross validation was conducted using random forest model implemented in randomForest package in R.

### 2.3 Ethical consideration

This study was approved by the ethics committees of the Research Institute National Center for Global Health and Medicine (approval number NCGM-A-003228), Nagasaki University (20191101), Tokai University (R19-075), Hokkaido University (2019-1), Hyogo College of Medicine (0419), and National Institute of Infectious Diseases (1283). Written informed consent was obtained from all participants.

### 2.4 Data availability

The RNA-Seq data have been deposited at NCBI GEO under accession number GSE187429. The matrix of TPM values summarized across the samples and intermediary files regarding the gene network are available at https://figshare.com/articles/dataset/A_matrix_of_TPM_values_obtained_from_whole_blood_RNA-Seq_data_of_11_CNO_patients_and_9_healthy_control_in_Japan/16843075

## 3 Results

**Fig. 2:**
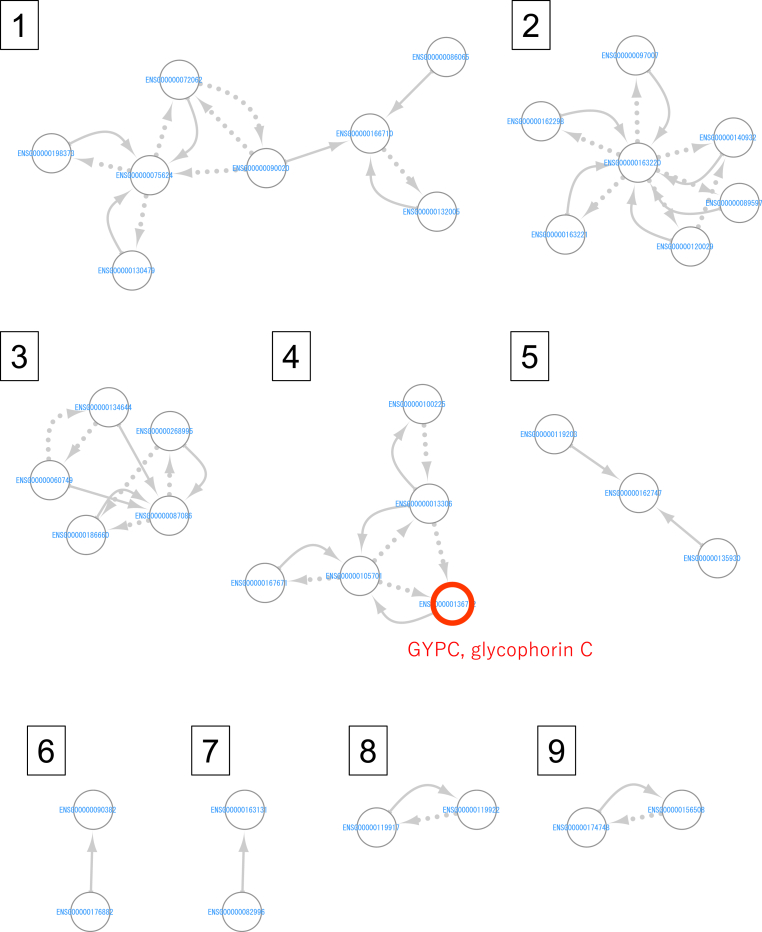
**Gene expression subnetwork characteristics of CNO.** Each circle represents a gene. The bold arrows indicate differentially regulated edges that are considered as CNO marker edges. The dashed arrows indicate other edges in the basal gene network estimated in the step of “Estimation of gene network structure” in Fig. 1. The red circles indicate the GYPC-encoding gene that showed the best discrimination ability as measured using AUC (Table 1). (For interpretation of the references to colour in this figure legend, the reader is referred to the Web version of this article.)

The amount of RNA-Seq data obtained for different samples varied from 6.1 to 7.9 Gb (Table S1), which were analyzed according to the workflow in Fig. 1. After preprocessing the data to prepare normalized read counts per gene as TPM and estimation of gene expression network structure, we identified differentially regulated edges that showed the top 0.001% highest difference in average ECv [6] in the CNO group compared to those in the healthy control group. ECv is useful in the extraction of group-specific subnetworks using a limited number of samples (e.g., 18 samples in a previous study [6]). In total, we identified nine subnetworks, including 26 differentially regulated edges (bold arrows in Fig. 2). The gene expression network not only captured the high correlation, but also estimated the direction of the relationship in terms of gene expression levels (indicated with arrows in Fig. 2).

The differentially regulated edges comprised 36 genes across nine subnetworks (Table 1). As the edges were not directly measurable, we analyzed the gene expression level calculated as TPM. In each subnetwork, the TPM values among the genes were highly correlated, and the absolute value of Pearson's correlation coefficient was at least 0.64. Thus, we identified a representative gene in each subnetwork that had the highest discrimination ability, indicated as “best AUC in each subnetwork” in Table 1. Using the nine representative genes, we constructed a model for discriminating patients with CNO and healthy controls and found that the best model included only a gene encoding glycophorin C (GYPC) (ENSG00000136732, marked as a red circle in Fig. 2); both classification tree model and automatic variable selection in the logistic regression model selected only this gene. We also explored whether accounting for potential interactions between the gene encoding GYPC and other representative genes could improve the discrimination between patients with CNO and healthy controls. We found that adding any other gene as an interaction term to the logistic regression model did not result in a decrease (i.e., improvement) of more than 1 of the Akaike information criterion, indicating the relative quality of statistical models.

**Table 1:** **Genes comprising the nine subnetworks that include the differentially regulated edges**.

subnetwork (Fig. 2)	gene ID	gene symbol	description	best AUC in each subnetwork
1	ENSG00000132005	RFX1	regulatory factor X1	0.76
	ENSG00000075624	ACTB	actin beta	
	ENSG00000166710	B2M	beta-2-microglobulin	
	ENSG00000072062	PRKACA	protein kinase cAMP-activated catalytic subunit alpha	
	ENSG00000090020	SLC9A1	solute carrier family 9 member A1	
	ENSG00000198373	WWP2	WW domain containing E3 ubiquitin protein ligase 2	
	ENSG00000086065	CHMP5	charged multivesicular body protein 5	
	ENSG00000130479	MAP1S	microtubule associated protein 1S	
2	ENSG00000140932	CMTM2	CKLF like MARVEL transmembrane domain containing 2	0.76
	ENSG00000163220	S100A9	S100 calcium binding protein A9	
	ENSG00000162298	SYVN1	synoviolin 1	
	ENSG00000097007	ABL1	ABL proto-oncogene 1, non-receptor tyrosine kinase	
	ENSG00000163221	S100A12	S100 calcium binding protein A12	
	ENSG00000089597	GANAB	glucosidase II alpha subunit	
	ENSG00000120029	ARMH3	armadillo like helical domain containing 3	
3	ENSG00000186660	ZFP91	ZFP91 zinc finger protein, atypical E3 ubiquitin ligase	0.81
	ENSG00000268995	VN1R82P	vomeronasal 1 receptor 82 pseudogene	
	ENSG00000134644	PUM1	pumilio RNA binding family member 1	
	ENSG00000060749	QSER1	glutamine and serine rich 1	
	ENSG00000087086	FTL	ferritin light chain	
4	ENSG00000136732	GYPC	glycophorin C (Gerbich blood group)	0.86
	ENSG00000013306	SLC25A39	solute carrier family 25 member 39	
	ENSG00000167671	UBXN6	UBX domain protein 6	
	ENSG00000100225	FBXO7	F-box protein 7	
	ENSG00000105701	FKBP8	FKBP prolyl isomerase 8	
5	ENSG00000119203	CPSF3	cleavage and polyadenylation specific factor 3	0.63
	ENSG00000162747	FCGR3B	Fc fragment of IgG receptor IIIb	
	ENSG00000135930	EIF4E2	eukaryotic translation initiation factor 4E family member 2	
6	ENSG00000090382	LYZ	lysozyme	0.61
	ENSG00000176882	AL135901.1	novel pseudogene	
7	ENSG00000082996	RNF13	ring finger protein 13	0.79
	ENSG00000163131	CTSS	cathepsin S	
8	ENSG00000119922	IFIT2	interferon induced protein with tetratricopeptide repeats 2	0.67
	ENSG00000119917	IFIT3	interferon induced protein with tetratricopeptide repeats 3	
9	ENSG00000156508	EEF1A1	eukaryotic translation elongation factor 1 alpha 1	0.60
	ENSG00000174748	RPL15	ribosomal protein L15	

To evaluate the discrimination ability of the gene encoding GYPC, we conducted leave-one-out cross validation using random forest model and found that sensitivity and specificity were 0.73 and 0.78, respectively. The main limitation of this discrimination analysis is the use of the same dataset for variable selection and evaluation of discrimination ability, and further studies, if possible, with larger samples, are warranted to use independent datasets for the selection and discrimination.

**Fig. 3:**
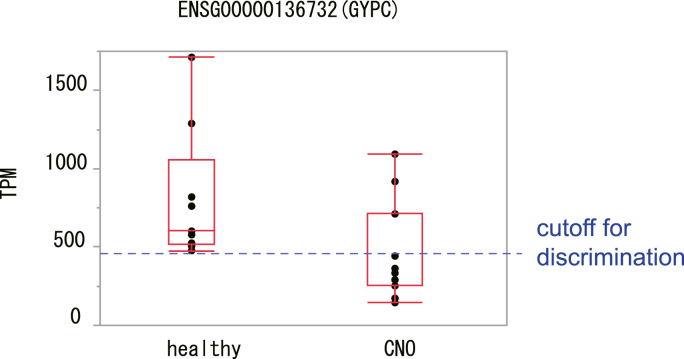
**Comparison of the expression levels of the CNO marker gene encoding GYPC between the healthy control and patients with CNO.** Y-axis: TPM. The blue dashed horizontal lines are cutoffs to achieve the best discrimination ability. (For interpretation of the references to colour in this figure legend, the reader is referred to the Web version of this article.)

The distribution of the TPM values of the gene encoding GYPC in the healthy control and CNO groups is shown as a box plot in Fig. 3. The TPM values were mostly lower in patients with CNO (median 333.7, interquartile range 252.3–711.7) than in the healthy controls (median 601.8, interquartile range 516.0–1054.2). The box plot suggests that the potential cutoff value of TPM for discriminating patients with CNO and healthy controls was approximately 450 (dashed blue line in Fig. 3).

## 4 Discussion

GYPC is an integral membrane glycoprotein that is broadly expressed in bone marrow and other tissues and plays an important role in regulating the mechanical stability of red cells [13]. It has a high level of Gerbich blood group antigens and is estimated to be present at 250,000 molecules per red cell [14]. In the membrane, GYPC comprises a part of the red cell junctional complex and is responsible for maintaining the lateral stability of the red cell membrane [14].

A decrease in GYPC levels is observed in cases of ovalocytosis [14], a rare hereditary red cell membrane defect characterized by the presence of oval erythrocytes. In the present study, the TPM values were mostly lower in patients with CNO than in the healthy controls (Fig. 3), which suggests abnormal red cell status in patients with CNO. In fact, approximately 40%–82% of patients with CNO reportedly have an elevated erythrocyte sedimentation rate [15], which is affected by the size or mass of red cells. Therefore, abnormality of red cells could be a characteristic of patients with CNO, although we could not confirm whether the abnormality of red cells is the primary factor that causes the disease or whether the abnormality is caused after the onset of the disease triggered by another factor.

**Fig. 4:**
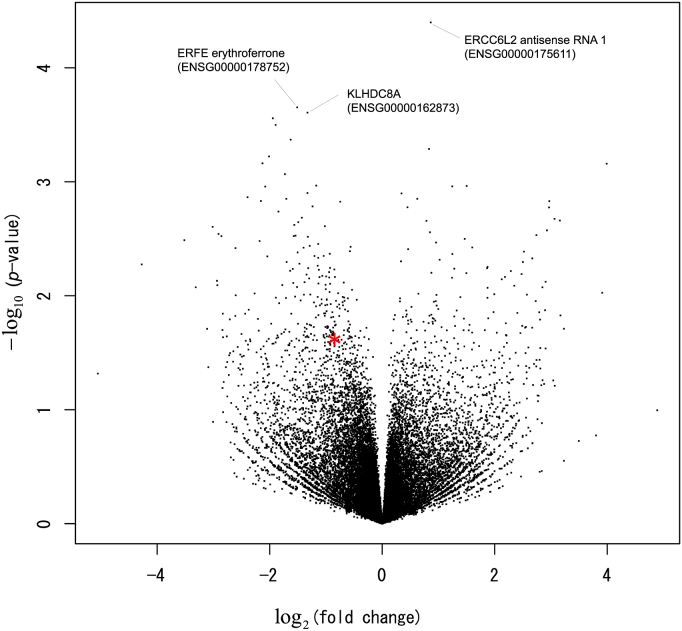
**Volcano plot showing the overall distribution of average fold changes and *P*-values among genes.** Genes with read counts higher than 6 are included. The key gene encoding GYPC is indicated as a red asterisk. Names of the 3 highest significant genes with the lowest *P*-values are also indicated at the top. (For interpretation of the references to colour in this figure legend, the reader is referred to the Web version of this article.)

The network-based approach that we utilized here to identify subnetworks including differentially regulated edges and genes can identify genes that cannot be identified using the traditional differential gene expression analysis [6]. In the previous study, 53% of genes identified using the network-based approach were not identified using the traditional method. Indeed, if the traditional DESeq2 package [16] is used to create a volcano plot showing the overall distribution of average fold changes and *P*-values among genes with read counts higher than 6 (Fig. 4), the key gene encoding GYPC was not identified as an outlier (red in Fig. 4). The key gene was a representative gene in the subnetwork underlying patient characteristics, which could not be captured using the traditional method.

The main limitation of this study is the small number of patients, in particular, the use of the same dataset for the selection of variables and evaluation of discrimination ability of GYPC. Our results should be validated using another dataset constructed through continuous sample collection, probably with two independent datasets for the selection of variables and evaluation of discrimination ability. Nonetheless, we consider this to be a valuable case study demonstrating the utilization of such a small sample dataset by applying a recently developed method that can extract a subnetwork of genes underlying patient characteristics.

In the present study, we conducted whole blood RNA-Seq exploiting the fact that peripheral blood is highly accessible and is a reliable indicator of human health. Compared to peripheral blood mononuclear cell samples, whole blood RNA preserved in PAXgene tubes, as in this study, has recently been reported to be excellent for producing gene expression data with minimal variability and good sensitivity [17]. However, for studies of whole blood RNA-Seq, managing high levels of hemoglobin mRNAs in blood, which impede the detection and quantification of non-hemoglobin RNAs, has been difficult [18]. Our procedure of RNA extraction and whole blood RNA-Seq is similar to that in a recent study that successfully identified disease-severity-specific neutrophil signatures useful for stratifying patients with COVID-19 (coronavirus disease 2019) [19]. To our knowledge, this is the first study to generate whole-blood RNA-Seq data (>6 Gb per sample) of 20 samples in Japan and make raw data and matrix of TPM values publicly available. From these data, we estimated, for the first time, a directed gene network, extracted the subnetwork of genes, and further identified the key gene underlying patient characteristics. The present study deepens our understanding of the rare disease CNO at the transcriptome level and further provides a framework for whole blood RNA-Seq and data analysis.

## Funding

This work was supported by 10.13039/501100001691JSPS Research Fellowships for Young Scientists to H.Y. (grant number 19J40070) and in part by Grants-in- Aid for Research from the 10.13039/100012319National Center for Global Health and Medicine (20A-3002 to H.Y.).

## Declaration of competing interest

The authors declare no conflict of interest.

## Data Availability

The RNA-seq data were deposited in NCBI GEO and the matrix of TPM values and intermediary files regarding the gene network are available as written in Data availability section
